# The intricate dance: host autophagy and *Coxiella burnetii* infection

**DOI:** 10.3389/fmicb.2023.1281303

**Published:** 2023-09-22

**Authors:** Tingting Wang, Chao Wang, Chang Li, Lei Song

**Affiliations:** ^1^Department of Infectious Diseases, First Hospital of Zibo City, Zibo, China; ^2^Department of Traditional Chinese Medicine, First Hospital of Zibo City, Zibo, China; ^3^Department of VIP Unit, China-Japan Union Hospital, Changchun, China; ^4^Department of Respiratory Medicine, Center for Pathogen Biology and Infectious Diseases, The First Hospital of Jilin University, Changchun, China

**Keywords:** Q fever, type IV secretion system, effector, autophagosome, lysosome

## Abstract

Q fever is a zoonotic disease caused by *Coxiella burnetii*, an obligatory intracellular bacterial pathogen. Like other intracellular pathogens, *C. burnetii* is able to survive and reproduce within host cells by manipulating host cellular processes. In particular, the relationship between *C. burnetii* infection and host autophagy, a cellular process involved in degradation and recycling, is of great interest due to its intricate nature. Studies have shown that autophagy can recognize and target intracellular pathogens such as *Legionella* and *Salmonella* for degradation, limiting their replication and promoting bacterial clearance. However, *C. burnetii* can actively manipulate the autophagic pathway to create an intracellular niche, known as the *Coxiella*-containing vacuole (CCV), where it can multiply and evade host immune responses. *C. burnetii* promotes the fusion of CCVs with lysosomes through mechanisms involving virulence factors such as Cig57 and CvpF. This review summarizes the latest findings on the dynamic interaction between host autophagy and *C. burnetii* infection, highlighting the complex strategies employed by both the bacterium and the host. A better understanding of these mechanisms could provide important insights into the development of novel therapeutic interventions and vaccine strategies against *C. burnetii* infections.

## 1. Introduction

*Coxiella burnetii*, known as the causative agent of Q fever, is an exceptionally infectious, intracellular bacterium that is regarded as a potential bioterrorism agent due to its highly contagious nature (Shaw and Voth, 2019). This gram-negative bacterium was first identified in the 1930s and was later named after Dr. Frank Burnet and Dr. Cox who were the first to describe it (Minnick and Raghavan, 2011). *C. burnetii* infection in humans ranges from asymptomatic to acute or chronic Q fever. Asymptomatic cases are common, and the infection is often discovered incidentally through serological testing. Acute Q fever is characterized by sudden onset of flu-like symptoms such as high fever, headache, muscle aches, fatigue, and cough (Ullah et al., 2022). The diagnosis is confirmed by serological testing, culture, and PCR (Ullah et al., 2022). In severe cases, acute hepatitis, pneumonia, or endocarditis may occur (Ullah et al., 2022). Chronic Q fever develops in less than 5% of infected individuals, usually in those with pre-existing heart valve defects, vascular disorders, or immunosuppression (Espana et al., 2020). Chronic Q fever can cause endocarditis, hepatitis, osteomyelitis, or vascular infections and requires prolonged antibiotic therapy and sometimes surgery (Espana et al., 2020).

Research on *C. burnetii* has increased in recent years, mainly due to its biodefense potential and its unique features as an intracellular pathogen. *C. burnetii* has a small genome (2.0 Mb) that encodes for approximate 1,800 proteins, including type IV secretion system (T4SS), lipopolysaccharides, and other virulence factors (Beare et al., 2006; Abou Abdallah et al., 2022). It can survive and replicate within the phagolysosome of macrophages and modulate host cell pathways to avoid immune recognition and clearance. *C. burnetii* also has the ability to form a dormant spore-like form, known as a small cell variant, which has increased virulence and persistence in host tissues (Sandoz et al., 2014). Understanding the molecular mechanisms of *C. burnetii* pathogenesis and host immune responses is crucial for developing effective treatment and prevention strategies.

## 2. Natural hosts of *Coxiella burnetii*

*Coxiella burnetii* is known to have a wide range of hosts, including mammals, birds, and arthropods, which serve as vectors for its transmission to human and animal populations (Celina and Cerny, 2022). Many different species of ticks, fleas, and lice have been identified as potential vectors for *C. burnetii*, and transmission also occurs through direct contact with infected animals or ingestion of contaminated food products (Celina and Cerny, 2022). Ticks are considered the primary vector host for *C. burnetii*, with the most common species being *Dermacentor variabilis* and *Rhipicephalus sanguineus* (Cabrera et al., 2022; Celina and Cerny, 2022). These ticks are known to feed on many different mammalian and avian hosts, and they can transmit *C. burnetii* to their hosts through their saliva. Fleas are another vector host for *C. burnetii*, with the most common species being *Xenopsylla cheopis*, which is known to transmit the bacterium to humans in regions where it is endemic (Eldin et al., 2017). Lice have also been identified as potential vector hosts for *C. burnetii*, although their role in transmission is not well understood (Eldin et al., 2017).

## 3. T4SS of *Coxiella burnetii*

One of the key virulence factors of *C. burnetii* is the T4SS. This complex apparatus is used by the bacterium to deliver effector proteins directly into the host cell cytoplasm. The T4SS of *C. burnetii* plays a crucial role in host-pathogen interactions and is a major determinant of the bacterium’s ability to cause disease (Cordsmeier et al., 2019; Thomas et al., 2020). The T4SS of *C. burnetii* is composed of multiple components, including the T4SS core complex, the coupling proteins, and the effector proteins. The T4SS core complex is the scaffold that supports the entire system, while the coupling proteins provide a bridge between the core complex and the effector proteins (Cordsmeier et al., 2019). The effector proteins of *C. burnetii*, similar to those of Legionella species (Song et al., 2021, 2022; Fu J. et al., 2022), play a crucial role as key virulence factors by being transported into the host cell cytoplasm. Once inside, they strategically manipulate host cell functions to promote bacterial survival and replication (Huang et al., 2022; Zhang et al., 2022).

## 4. Host autophagy in *Coxiella burnetii* infection

Autophagy is a highly regulated process that involves the formation of double-membraned vesicles called autophagosomes, which sequester intracellular components and deliver them to the lysosomes for degradation (Aman et al., 2021). This process is crucial for maintaining cellular homeostasis and plays a role in various physiological and pathological conditions, including bacterial infections (Yuk et al., 2012; Aman et al., 2021). Several mechanisms by which autophagy influences bacterial infections have been identified. One important aspect is the selective targeting of bacteria by autophagy. Upon infection, autophagy can recognize and engulf intracellular bacteria within autophagosomes, leading to their delivery to lysosomes for degradation (Sharma et al., 2018). This process restricts bacterial replication and spread, thereby limiting the severity of the infection. Examples of bacterial pathogens targeted by autophagy include *Salmonella*, *Legionella*, *Mycobacterium tuberculosis*, and Group A *Streptococcus* (Sharma et al., 2018; Thomas et al., 2020).

It is interesting to note that the relationship between host autophagy and *C. burnetii* infection is quite distinct from that of other pathogens mentioned above. The *Coxiella*-containing vacuole (CCV) formed during *C. burnetii* infection accumulates markers commonly found in autophagosomes and lysosomes such as Rab7 (Beron et al., 2002), LC3 (Gutierrez et al., 2005), Beclin 1 (Vazquez and Colombo, 2010) and LAMP-1/2 (Schulze-Luehrmann et al., 2016). Research has indicated that the stimulation of host autophagy, whether through nutrients deprivation or the administration of rapamycin, results in a rise in *C. burnetii* replication, viability, and CCV size (Gutierrez et al., 2005), whereas inhibiting autophagy or preventing vacuole acidification impairs CCV development (Beron et al., 2002; Latomanski and Newton, 2018). Essential autophagy genes, such as TFEB/TFE3, autophagy-related (ATG) proteins, and STX17, are required for the homotypic fusion process, resulting in the formation of large CCVs (Mcdonough et al., 2013; Newton et al., 2020; Padmanabhan et al., 2020; Lau et al., 2022). Therefore, *C. burnetii* benefits from the autophagy process and facilitates autophagosome-lysosome fusion to establish its distinct parasitophorous niche for replication.

## 5. Host autophagy manipulation by T4SS substrates

Given the critical role of autophagy in facilitating infection, it is unsurprising that *C. burnetii* has developed intricate mechanisms to exploit and manipulate the host’s autophagic machinery for its own benefit. Romano et al. (2007) reported that *C. burnetii* infection resulted in a postponement of the usual arrival of cathepsin D, a lysosomal protease, to the CCV, which was further extended by autophagy induction by starvation. Under normal circumstances, the cargo receptor p62/sequestosome 1 (SQSTM-1) and LC3 interact to facilitate the selection of cargo for degradation through autophagy, leading to the degradation of p62 and recycling of LC3 (Zhou et al., 2023). The levels of p62 and LC3 were increased in macrophages in response to *C. burnetii* infection, implying that the pathogen plays a role in stabilizing the proteins (Winchell et al., 2014, 2018; Larson et al., 2019). Furthermore, several genes involved in autophagy-related pathways, specifically membrane trafficking and lipid metabolism, were upregulated in *C. burnetii* Nine Mile phase II (NMII) infected cells, while they were downregulated in NMI-infected cells (Kumaresan et al., 2022).

The mounting evidence suggests that the functional type IV secretion system (T4SS) of *C. burnetii* is essential for manipulating host autophagy. LC3 and p62 were present in the wild-type CCVs but not in CCVs containing T4SS mutants of *C. burnetii* (Winchell et al., 2014). In addition, upon activation of the T4SS 24 h post-infection, there was a subsequent recruitment of autophagosomes (Winchell et al., 2014). TFEB and TFE3 were both activated upon *C. burnetii* infection, as indicated by their movement from the cytoplasm to the nucleus (Padmanabhan et al., 2020). The movement of the transcription factors was discovered to depend on the T4SS, as the nuclear movement of TFEB and TFE3 was reduced in a Dot/Icm mutant (Padmanabhan et al., 2020).

### 5.1. *Coxiella* vacuolar protein B (CvpB)

CvpB, also known as Cig2, is one of the key effectors delivered by the T4SS of *C. burnetii*. CpvB plays a crucial role in the modulation of host immune responses and the establishment of a favorable intracellular niche for *C. burnetii* replication. Studies have shown that CpvB is involved in multiple aspects of *C. burnetii* infection. One of its important functions is interfering with host autophagy. Newton, et al. identified mutants displaying a multi-vacuolar phenotype, all of which had a transposon insertion in the gene encoding the effector protein CvpB (Newton et al., 2014). In comparison to vacuoles containing wild-type *C. burnetii*, the vacuoles formed by the *cvpB* mutant lacked detectable amounts of the autophagosome protein LC3 (Newton et al., 2014). Interestingly, when host autophagy was disrupted during infection by wild-type *C. burnetii*, a similar multi-vacuolar phenotype was observed, resembling that of the *cvpB* mutant (Newton et al., 2014). CvpB, as well as CvpC, CvpD, and CvpE, label the CCV membrane and LAMP1-positive vesicles and deletion mutants for any of these substrates show impaired intracellular replication and CCV formation (Larson et al., 2015). Furthermore, CvpB is crucial for facilitating continuous fusion between the CCV and autophagosomes produced through selective autophagy (Kohler et al., 2016). This interaction leads to the formation of an autolysosomal vacuole (Kohler et al., 2016). These findings strongly suggest that the functional CvpB protein plays a vital role in the interaction between the CCV and host autophagosomes.

Another study has reported that CpvB interacts with PI(3)P and phosphatidylserine (PS) on CCVs and early endosomes (Martinez et al., 2016). CvpB disrupts the activity of a phosphatidylinositol 5-kinase called PIKfyve, which plays a crucial role in regulating PI(3)P metabolism (Martinez et al., 2016). Within CCVs, CvpB’s association with PI(3)P derived from early endosomes and autophagy pathways, along with the inhibition of PIKfyve, enhances the association of the autophagosomal machinery with CCVs (Martinez et al., 2016). However, the molecular mechanisms by which CvpB prevents PIKfyve recruitment to PI(3)P-positive membranes remain unknown. Further research is required to elucidate the exact mechanisms underlying these observations and to fully understand the role of CvpB in manipulating PI(3)P metabolism, autophagy and CCV biogenesis.

### 5.2. *Coxiella* vacuolar protein F (CvpF)

CvpF belongs to the Cvp family of effectors, which are secreted by the Dot/Icm secretion system of *C. burnetii*. Upon screening a *C. burnetii* transposon mutant library, researchers found that mutations in the *cvpF* gene resulted in defects in intracellular replication and vacuole formation (Siadous et al., 2021). CvpF localizes to vesicles with autolysosomal characteristics, as well as CCVs. CvpF was found to interact specifically with the host protein RAB26, a small GTPase involved in regulating endosomal trafficking (Siadous et al., 2021). This interaction leads to the recruitment of MAP1LC3B/LC3B, a marker protein for autophagosomes, to the CCVs (Siadous et al., 2021). Importantly, studies using *cvpF*::Tn mutants showed that these mutants were highly attenuated and less virulent compared to wild-type *C. burnetii* in a mouse model of infection (Siadous et al., 2021). This highlights the significance of CvpF in *C. burnetii*’s ability to cause disease and establish infection within the host.

### 5.3. *Coxiella* plasmid effector B (CpeB)

Another *C. burnetii* effector protein CpeB, which is encoded by the QpH1 plasmid, was found to localize on the membrane of the CCVs and interact with LC3 and LAMP1, respectively (Voth et al., 2011). This interaction promotes the accumulation of LC3-II, a lipidated form of LC3 that is associated with autophagosomal membranes (Fu M. et al., 2022). When the QpH1 plasmid was absent in *C. burnetii* strains, there was reduced LC3-II accumulation, smaller CCVs, and lower bacterial loads in host cells (Fu M. et al., 2022). Furthermore, expression of CpeB in the QpH1-deficient strain restored LC3-II accumulation, indicating that CpeB plays a significant role in promoting LC3-II accumulation (Fu M. et al., 2022). The study also found that CpeB targets a host protein called Rab11a to promote LC3-II accumulation (Fu M. et al., 2022). Rab11a is involved in regulating membrane trafficking pathways, including those associated with autophagy. When Rab11a was conditionally knocked out in mice and subsequently infected with *C. burnetii*, notable reductions in bacterial burdens and the development of milder lung lesions were observed (Fu M. et al., 2022), suggesting that Rab11a plays a role in *C. burnetii* infection and virulence.

### 5.4. Others

Apart from the aforementioned proteins, such as CpeB, additional *C. burnetii* effector proteins like Cig57, CvpA, CvpC, and others are believed to contribute to the intricate interplay between *C. burnetii* infection and autophagy (Thomas et al., 2020).

## 6. Conclusion and perspective

In conclusion, *C. burnetii* has a sophisticated set of tactics to subvert the host autophagic pathways, allowing it to survive and thrive within host cells. The bacterium’s ability to manipulate this innate cellular process and evade immune detection contributes to its pathogenesis in humans and animals. Looking forward, it is important to note that there are still gaps in our understanding of *C. burnetii*’s subversion of autophagy, particularly in identifying and characterizing autophagy-related effectors with explored biochemical activities. Future research should aim to fill these knowledge gaps by further investigating the specific virulence factors involved in *C. burnetii*’s manipulation of autophagy. This research may provide valuable insights into potential targets for antimicrobial interventions. Additionally, exploring strategies to modulate the host immune response to *C. burnetii* infections could enhance immune clearance of the bacterium. Ultimately, a comprehensive understanding of the host-pathogen interactions and the biochemical activities of autophagy-related effectors will be crucial for developing effective treatments and control measures to combat *C. burnetii* infections.

## Author contributions

TW: Conceptualization, Writing – original draft, Writing – review & editing. CW: Conceptualization, Writing – original draft. CL: Conceptualization, Writing – review & editing. LS: Conceptualization, Funding acquisition, Writing – original draft, Writing – review & editing.
