# Supplementary material for: The intricate dance: host autophagy and Coxiella burnetii infection
Source: Front Microbiol. 2023 Sep 22;14:1281303. doi: 10.3389/fmicb.2023.1281303 (PMC10556474; doi:10.3389/fmicb.2023.1281303)
Supplement: Supplementary file 1 [file Table_1.DOCX]

**TABLE 1 *C. burnetii* T4SS effectors involved in host autophagy.**

| **Effectors (Gene ID)** | **Aliases** | **Interactor/Substrate** | **Enzymatic activity** | **Function** | **References** |
| --- | --- | --- | --- | --- | --- |
| **CBU_0021** | CvpB/ Cig2 | PIKfyve | Unknown | Regulation of PI(3)P metabolism | Newton *et al*., 2014; Larson *et al*., 2015; Kohler *et al*., 2016; Martinez *et al*., 2016 |
| **CBU_0626** | CvpF | RAB26 | Unknown | Recruitment of LC3B to CCVs | Siadous *et al*., 2021 |
| **CBUA0013** | CpeB | Rab11a | Unknown | accumulation of LC3-II | Fu et al., 2022 |
| **CBU****_1751** | Cig57 | FCHO2 | Unknown | Recruitment of Clathrin to CCVs | Newton *et al*., 2014; Latomanski *et al*., 2016; Siadous *et al*., 2021 |
| **CBU_0665** | CvpA | Clathrin adaptor protein subunit 2 and Clathrin heavy chain | Unknown | engagement of the endocytic recycling system | Larson *et al*., 2013 |
| **CBU_1556** | CvpC/Cig50 | Unknown | Unknown | CCVs development | Larson *et al*., 2015 |
